# Colorimetric aptasensing of microcystin-LR using DNA-conjugated polydiacetylene

**DOI:** 10.1007/s00216-024-05617-x

**Published:** 2024-10-28

**Authors:** Man Zhang, Qicheng Zhang, Lei Ye

**Affiliations:** Division of Pure and Applied Biochemistry, Department of Chemistry, Lund University, 22100 Lund, Sweden

**Keywords:** Polydiacetylene, Microcystin-LR, DNA aptamer, Colorimetric detection

## Abstract

Polydiacetylene (PDA) holds promise as a versatile material for biosensing applications due to its unique optical properties and self-assembly capabilities. In this study, we developed a colorimetric detection biosensor system utilizing PDA and aptamer for the detection of microcystin-LR (MC-LR), a potent hepatotoxin found in cyanobacteria-contaminated environments. The biosensor was constructed by immobilizing MC-LR-specific aptamer on magnetic beads, where the aptamer was hybridized with a urease-labelled complementary DNA (cDNA-urease). Upon binding MC-LR, the aptamer undergoes a conformational change to release cDNA-urease. The released cDNA-urease is subsequently captured by PDA bearing a single-stranded DNA (ssDNA). The enzymatic reaction triggers a distinctive color transition of PDA from blue to red. The results demonstrate exceptional sensitivity, with a linear detection range of 5–100 ng/mL and a limit of detection as low as 1 ng/mL. The practicability of the colorimetric method was demonstrated by detecting different levels of MC-LR in spiked water samples. The recoveries ranged from 77.3 to 102% and the color change, visible to the naked eye, underscores the practical utility for on-site applications. Selectivity for MC-LR over other microcystin variants (MC-RR and MC-YR) was confirmed. The colorimetric detection platform capitalizes on the properties of PDA and nucleic acid, offering a robust method for detecting small molecules with potential applications in environmental monitoring and public health.

**Graphical Abstract:**

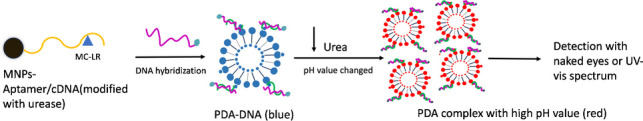

**Supplementary Information:**

The online version contains supplementary material available at 10.1007/s00216-024-05617-x.

## Introduction

Microcystin-LR (MC-LR), a potent hepatotoxin produced by certain strains of cyanobacteria such as *Microcystis aeruginosa*, poses significant environmental and public health risks due to its high toxicity and widespread occurrence in freshwater systems [1, 2]. This toxin is one of the most studied microcystin variants, primarily because of its severe impact on human and animal health [3]. Exposure to MC-LR can lead to liver damage and promote hepatocellular carcinoma by inhibiting protein phosphatases 1 and 2A, which are critical for cellular function and signaling pathways [4]. The persistence and resilience of MC-LR in aquatic environments are exacerbated by its chemical stability and resistance to degradation, even under varying environmental conditions [5]. This stability allows MC-LR to accumulate in water bodies, bioaccumulate in aquatic organisms, and enter the food chain, posing a chronic threat to human health [6]. Therefore, it is crucial to develop effective, inexpensive, and uncomplicated methods for the detection of MC-LR. Traditional methods such as enzyme-linked immunosorbent assays (ELISA) [7] and equipment-based techniques [8], although effective, are often limited by high costs and complexity. Recently, colorimetric detection strategies for MC-LR, based on enzymes like horseradish peroxidase (HRP) [9] and nanomaterial-based artificial enzymes [10], have been reported. However, there is a need for a new colorimetric method that can provide rapid, sensitive, and specific detection of MC-LR.

An effective strategy for achieving selective detection of MC-LR is the use of aptamers, which are short, single-stranded DNA or RNA molecules that can specifically bind to target analytes with high affinity. Aptamers offer several advantages over traditional antibodies, including better stability, ease of chemical modification, and lower production costs. In this study, we employed an MC-LR-specific DNA aptamer with an affinity constant (K_d_) of 50 ± 12 nM [11], ensuring a strong interaction with MC-LR even at low concentrations. This high specificity is critical for distinguishing MC-LR from other structurally similar microcystin variants, such as microcystin-RR (MC-RR) and microcystin-YR (MC-YR), reducing the risk of cross-reactivity.

Polydiacetylene (PDA), a conjugated polymer known for its unique optical properties, has emerged as a versatile platform for biosensing applications. Upon exposure to UV light (254 nm), PDA undergoes a topochemical polymerization, resulting in a chromatic transition [12, 13] from blue to red in response to various stimuli, such as heat [14], mechanical stress [15], pH variation [16, 17], and binding of biomolecules [18, 19]. This chromatic transition makes PDA an ideal material for biosensing, as its color change can be observed visually without the need for sophisticated detection equipment. Colorimetric sensors using the special optical characteristics of PDA have been developed in various fields and successfully applied to the visual detection of viruses [20, 21], chemicals [22, 23], arteriosclerosis [24], and platelets [25]. Current research on PDA and functional nucleic acids follows two main models: modifying nucleic acid probes on PDA surfaces to cause aggregation and color change upon target interaction and synthesizing positively charged PDA particles to leverage ionic interactions with nucleic acids [26, 27]. For example, Jung et al. introduced a colorimetric approach using PDA particles for nucleic acid detection, which was later refined for enhanced specificity [28]. Zhou et al. integrated a *Bacillus*-specific aptamer with PDA for detecting *Bacillus thuringiensis* on a paper device [29]. However, interactions based on nucleic acid-to-nucleic acid or ionic interactions often induce limited color changes in PDA. Additionally, PDA-based nucleic acid sensing methods are currently limited to detecting large molecules. This limitation underscores the need for further research to enhance the versatility and specificity of PDA-based nucleic acid for small molecule detection.

The versatility of PDA as a biosensing material is further enhanced by its sensitivity to pH changes, which underpins its chromatic transition mechanism. This pH-responsive behavior is crucial for applications where environmental or biochemical changes induce local pH shifts. The intrinsic mechanism of PDA’s pH-responsive behavior is particularly important for its use in biosensors. PDA’s transition from blue to red is intrinsically linked to the protonation state of functional groups on its polymer backbone. In neutral to mildly basic conditions (pH 7.0–8.0), the PDA chains maintain a highly ordered structure, resulting in a blue color due to π-π conjugation along the backbone. However, when the pH increases beyond approximately 8.5, deprotonation of these functional groups occurs, disrupting the molecular ordering within the polymer. This disruption reduces the effective conjugation length, causing a shift in the absorption spectrum from blue (650 nm) to red (550 nm).

By integrating PDA nanoparticles with an MC-LR-specific aptamer, we aim to develop a colorimetric biosensor that combines the specificity of aptamer recognition with the pH-responsive behavior of PDA. The conformational change in the aptamer upon binding to MC-LR triggers the release of cDNA-urease, which subsequently catalyzes the hydrolysis of urea. This enzymatic reaction raises the local pH, inducing the blue-to-red transition in the PDA nanoparticles. This color change can be observed by the naked eye, providing a rapid and easily interpretable signal without the need for complex instrumentation. For more quantitative analysis, the color change can also be measured using a UV–vis spectrophotometer, offering a robust method for monitoring MC-LR concentrations in environmental samples.

## Experimental section

### Materials and equipment

The oligonucleotides used in this work (Table S1) were obtained from Sangon Biotech Co., Ltd. (Shanghai, China). Microcystins, including microcystin-YR (MC-YR) and microcystin-RR (MC-RR), were provided by ENZO Life Sciences (Germany). 10,12-Pentacosadiynoic acid (PCDA), 1,2-dimyristoyl-sn-glycero-3-phosphocholine (DMPC), *N*-hydroxysuccinimide (NHS), *N*-ethyl-*N*′-(3-dimethylaminopropyl) carbodiimide hydrochloride (EDC), urease, and urea were obtained from Sigma-Aldrich (Sweden). Dynabeads™ M-280 streptavidin was purchased from Thermo Fisher Scientific Co., Ltd. (USA). All chemical reagents were of analytical grade and used as received.

A microplate reader (Thermo Fisher Scientific Co., Ltd., Sweden) was used to measure and collect UV–vis absorption spectra. A dynamic light scattering instrument (Zetasizer Nano ZS, Malvern Instruments, UK) was employed to measure zeta potential and particle size. SEM was carried out using JEOL JSM 6700F scanning electron microscope (Japan).

### Synthesis and characterization of PCDA-NHS monomer

PCDA-NHS was synthesized based on a reported method with slight modifications [30]. PCDA (1 g, 2.7 mmol), NHS (460 mg, 4 mmol), and EDC (620 mg, 4 mmol) were dissolved in 20 mL of dichloromethane (DCM) and stirred for 4 h at room temperature. The solvent was evaporated using a rotary evaporator. The resulting crude product was dissolved in water and purified by extracting three times with ethyl acetate. The organic layer was dried using magnesium sulfate, and the ethyl acetate was removed by rotary evaporation. The PCDA-NHS monomer was obtained as a white powder (550 mg). The product was characterized using Fourier transform infrared (FTIR) spectroscopy and proton nuclear magnetic resonance spectroscopy. 1H NMR (400 MHz, DMSO-d_6_: *d* 0.86 (t, *J* = 6.8 Hz, 3 H), 1.24–1.32 (m, 26 H), 1.41–1.46 (m, 4 H), 1.60–1.63 (m, 2 H), 2.26 (t, *J* = 8.0 Hz, 4 H), 2.64 (t, *J* = 8.0 Hz, 2 H), 2.81 (s, 4 H) (Fig. S1).

### Synthesis of PDA-DNA

The DNA functionalized PDA was prepared based on a previous method with slight modifications.^26^ In brief, PCDA (18.73 mg), PCDA-NHS (4.71 mg), and DMPC (27.11 mg) were dissolved in chloroform in a molar ratio of 5:1:4, respectively, to a total volume of 10 mL. The solvent was then removed by purging with nitrogen gas (N₂). The residue was redissolved in 10 mL of Milli-Q (MQ) water and sonicated at 80 °C for 20 min. The solution was filtered through a 0.45-μm pore membrane to control the particle size, resulting in a final particle concentration of 10 mM. Next, 1 nmol of NH₂-DNA was added to the lipid solution, which was then shaken at 37 °C for 4 h. Subsequently, 1 μL of ethanolamine (100 mM) was added and the mixture was incubated for an additional 2 h to block any un-reacted NHS groups. The solution was stored at 4 °C for at least 8 h (overnight) to complete the self-assembly of the lipid particles. The product was irradiated with UV light at 254 nm for 20 min to give the final PDA nanoparticles.

### Conjugation of NH_2_-DNA with urease

Maleimidobenzoic acid *N*-hydroxysuccinimide ester (MBS) was used to conjugate a 5′-amino-modified DNA oligonucleotide (NH₂-DNA) to urease. Initially, NH₂-DNA was reacted with MBS to produce maleimidobenzoic DNA amide (MDNA). Urease was then coupled to MDNA via the reaction of the thiol group of cysteine to the double bond of the maleimide in MDNA.

First, a solution of MBS was prepared by dissolving 2 mg of MBS (6.4 μmol) in 1 mL of DMSO. Then, 1.5 mg of urease (3.3 nmol) was dissolved in 1 mL of 1 × PBS buffer (pH 7.2). Next, 1 nmol of NH₂-DNA and 3.2 μL of the MBS solution (20 nmol) were mixed well in 1 × PBS buffer to a final volume of 100 μL and incubated at room temperature for 1 h. Excess MBS was removed by passing through a 3-kDa molecular weight cutoff filter in a centrifuge column. The activated DNA was washed three times with 1 × PBS buffer and redissolved in 100 μL of 1 × PBS buffer.

Subsequently, 1 mL of the urease solution (3.3 nmol) was added to the MBS-activated DNA and incubated at room temperature for 1 h. The mixture was filtered through a 300-kDa molecular weight cutoff filter in a centrifuge column to remove the excess urease and un-conjugated DNA. Finally, the DNA-urease conjugate was washed three times with 50 μL of 1 × PBS buffer and redissolved in 100 μL of 1 × PBS buffer.

### Preparation of MB probe and establishment of detection system

#### Preparation of MB-aptamer probe

A total of 200 μL of streptavidin-coated magnetic beads (MBs) (10 mg/mL) was washed twice with 1 × binding and washing buffer (2 mM Tris–HCl, 1 M NaCl, 0.5 mM EDTA, pH 7.5) and resuspended in 400 μL of the same buffer. Subsequently, 400 μL of biotin-aptamer (2 μmol/L) was added to the beads and incubated at room temperature for 15 min. The MB-aptamer complex was then washed serially with 1 × binding buffer, washing buffer, and water to remove the excess aptamer. Finally, the MB-aptamer complex was redispersed in 200 μL of water.

#### Synthesis of MB-aptamer/cDNA-urease complex

To synthesize the MB-aptamer/DNA-urease complex, 400 μL of MB-aptamer (2 μM) was mixed with 100 μL of cDNA-urease (approximately 10 μM) and incubated at room temperature for 30 min. The complex was then washed three times with water and resuspended in 500 μL of water.

#### Detection system

Different concentrations of MC-LR (0, 0.1 ng/mL, 0.5 ng/mL, 1 ng/mL, 5 ng/mL, 10 ng/mL, 20 ng/mL, 40 ng/mL, 50 ng/mL, 80 ng/mL, and 100 ng/mL) were prepared in 100-μL aliquots. Each aliquot was mixed with 100 μL of the MB-aptamer/DNA-urease complex and incubated on a rocking table at room temperature for 30 min. The supernatant, containing different concentrations of DNA-urease, was collected using magnetic separation. To each 50-μL supernatant, 100 μL of PDA-DNA and 100 μL of urea (100 mM) were added and incubated at room temperature for 1 h. The mixtures were then analyzed using a microplate reader. Absorbance profiles were recorded, and data were quantitatively analyzed using the colorimetric response (CR) (%) equation. Data from three independent experiments were collected, and each plotted point represents the mean value with error bars indicating the standard deviation.

### Selectivity and real sample test

The selectivity of our biosensor was evaluated by preparing solutions of MC-LR, MC-RR, MC-YR, and a mixture of these three microcystins, each at a final concentration of 50 ng/mL. These toxin solutions were mixed with the magnetic bead-aptamer/DNA-urease complex, allowing for specific binding. After a defined incubation period, the supernatant was collected for further analysis. This mixture was then incubated with the PDA-DNA nanoparticles and urea at room temperature for 1 h. The UV absorption spectrum was subsequently measured to assess the colorimetric response.

To further validate the practical applicability of our biosensor, we conducted tests using real water samples. Tap water was collected directly from the water system in the Chemical Center at Lund University. After a consistent flow for at least 1 min, the tap water was taken and filled in a clean 10-mL polyethylene bottle. River water was collected from a river in the north of Lund. The sample was collected in the middle of the stream, at a depth of approximately 20 cm to avoid surface debris. After the collection, all samples were immediately sealed and transported in a cooler with ice packs to maintain a temperature of 4 °C, preserving the integrity of microcystins. Prior to the laboratory tests, the samples were filtered through a 0.45-μm syringe filter to remove particulate matter, followed by spiking with known concentrations of MC-LR (10 ng/mL, 50 ng/mL, and 100 ng/mL) to simulate contamination scenarios. The subsequent steps followed the procedure as described above.

## Results and discussion

### Principle of MC-LR detection using PDA-DNA and magnetic-aptamer

**Scheme 1:**
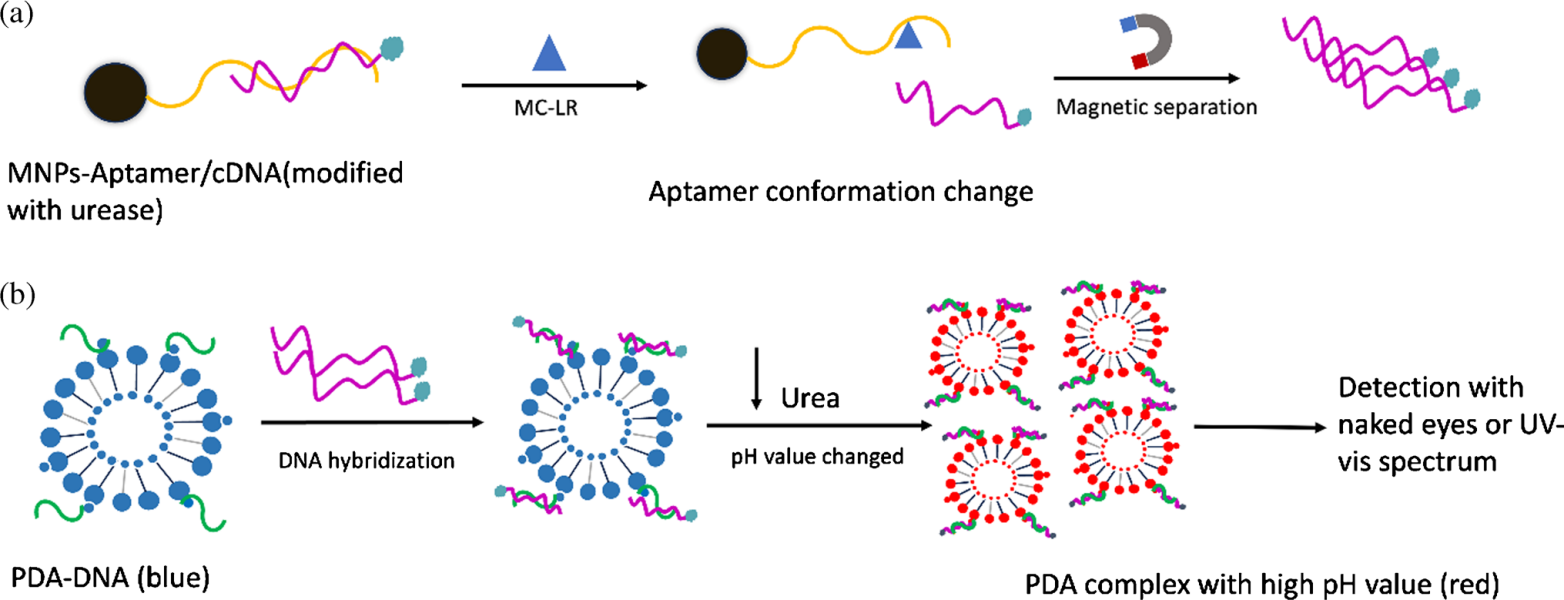
Principle of the analytical system using polydiacetylene-DNA and magnetic-aptamer for colorimetric detection of microcystin-LR

The principle of detecting MC-LR using the PDA-DNA and magnetic-aptamer is illustrated in Scheme 1. Part A: Magnetic beads are conjugated with an MC-LR aptamer and a complementary DNA-urease (cDNA-urease). The aptamer is attached on magnetic bead via a streptavidin–biotin linkage. Upon exposure to MC-LR, the aptamer undergoes a conformational change, releasing the cDNA-urease. After magnetic separation, the released cDNA-urease is collected for subsequent steps. Part B: PCDA, DMPC, and NHS-activated diacetylene monomer (PCDA-NHS) are co-assembled in water to form nanoparticles. The amine-functionalized binding DNA, which is complementary to the DNA sequence in the cDNA-urease, is covalently attached to the diacetylene particles via NHS/EDC-mediated coupling reaction. The final functionalized PDA-DNA nanoparticles are obtained through photo-induced polymerization. For analytical quantification, the cDNA-urease released in Part A is captured by the PDA-DNA nanoparticles through a DNA hybridization reaction. The urease catalyzes the hydrolysis of urea, increasing the system’s pH and inducing a blue-to-red color change of the PDA nanoparticles. This colorimetric signal can be detected either visually or by using a UV–vis microplate reader.

### Characterization of DNA-functionalized PDA nanoparticles (PDA-DNA)

**Fig. 1:**
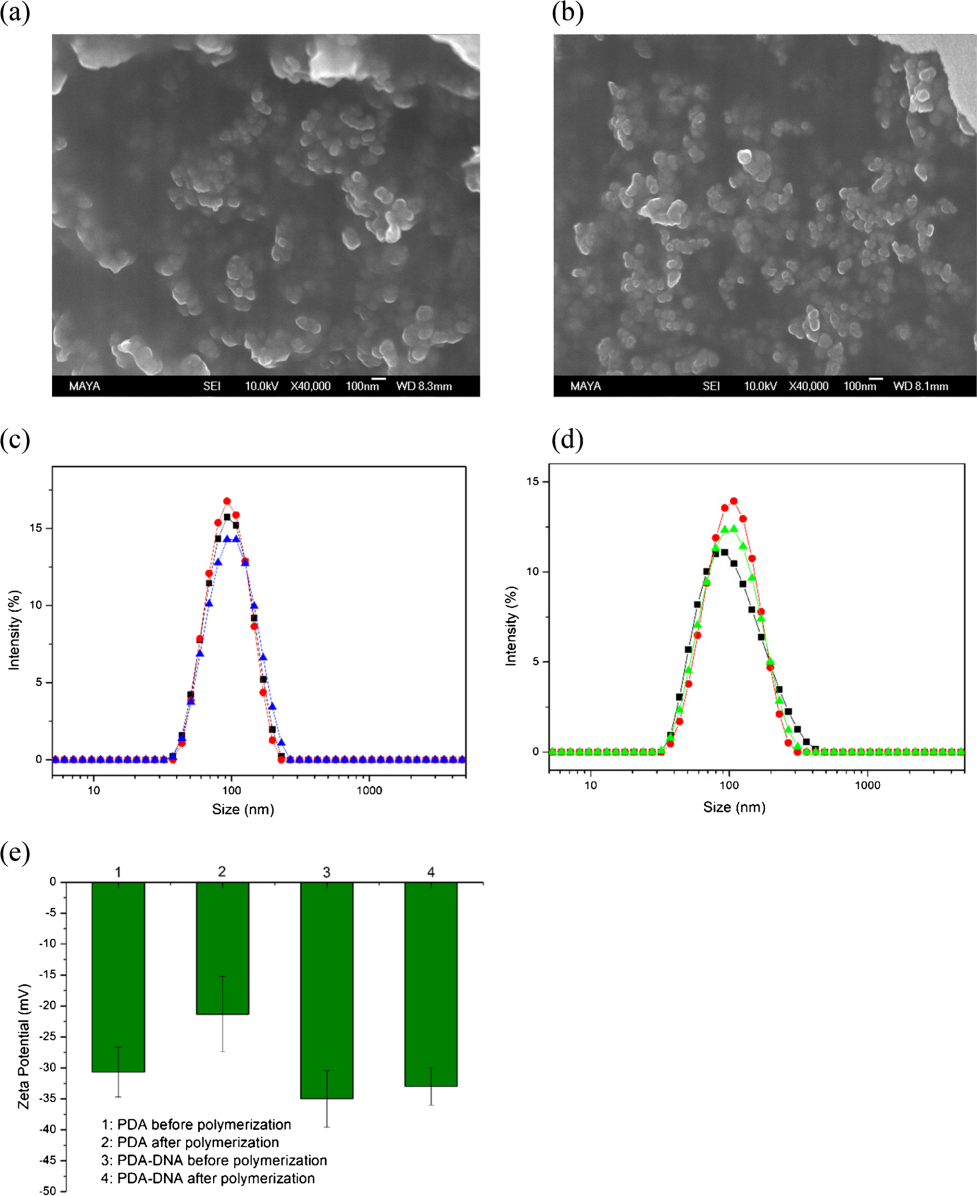
**a**, **b** SEM images of PDA-DNA particles before and after polymerization. Scale bar: 100 nm. **c**, **d** Size distribution of PDA-DNA before and after polymerization. **e** Zeta potential of PDA without DNA modification and PDA-DNA particles before and after polymerization

As shown in Fig. 1a, b, the DNA-functionalized diacetylene particles displayed an ellipsoid-like structure before and after polymerization, as confirmed by scanning electron microscopy (SEM). The size of the particles changed from 100 to 150 nm, as shown in Fig. 1c, d, which can be attributed to the formation of the conjugated enyne backbone during the polymerization. It is noteworthy that these morphological and dimensional changes are similar to PDA nanoparticles prepared without the DNA modification (Fig. S2), implying that DNA conjugation does not influence the structural properties of PDA nanoparticles; rather, it enhances their functional capabilities. To confirm the successful fabrication of the nanoprobe, the zeta potential was recorded, as shown in Fig. 1e. The zeta potential measurements further elucidate the surface charge dynamics of the nanoparticles, with values indicating successful conjugation of negatively charged oligonucleotides. Specifically, the zeta potentials of unmodified PDA particles shifted from − 30.6 to − 21.3 mV after polymerization, and subsequently decreased to − 35 mV for PDA-DNA particles. This increase in negativity is crucial, as it not only stabilizes the nanoparticles in solution but also facilitates their interaction with target analytes.

A 12% polyacrylamide gel electrophoresis (PAGE) assay was performed to further verify the coupling of DNA with PDA (Fig. S3). Free DNA in lane 2 showed a single band after electrophoresis, while PDA-DNA in lane 1 displayed slow-moving band with slight tailing. This slow-moving can be attributed to the coupling of DNA with PDA, which hinders the migration of DNA in the crosslinked gel. Additionally, energy-dispersive X-ray spectroscopy (EDS) was used to confirm the conjugation of DNA with PDA (Fig. S4). The EDS results confirmed the presence of phosphorus element in the PDA-DNA sample. All these results indicate the successful conjugation of DNA with PDA.

### Verification of DNA-urease conjugation

The conjugation of DNA-urease was verified using 12% PAGE. As shown in Fig. S5, lane 1 contains the purified urease conjugated with NH₂-DNA, displaying an intense band at the top of the gel. Lane 2 contains a mixture of urease and NH₂-DNA, showing a DNA band below 50 bp. Lane 3 contains urease alone, with no visible DNA band. These results confirm the successful coupling of NH₂-DNA with urease. To determine the conjugation efficiency, we measured the urease concentration after the conjugation reaction using a bicinchoninic acid assay (BCA) protein quantification. Based on the protein standard curve (Fig. S6), we calculated the concentration of urease-DNA to be 0.554 mg/mL. Therefore, the efficiency of the urease-DNA conjugation is 37%. The urease-DNA conjugate is of paramount importance, as it directly influences the biosensor’s enzymatic activity and overall performance in MC-LR detection.

### Detection of MC-LR using PDA-DNA and the aptamer system

According to the principle illustrated in Scheme 1, the binding of MC-LR to the aptamer on the magnetic bead is expected to cause the cDNA-urease to dissociate from the aptamer. The released cDNA-urease then catalyzes hydrolysis of urea and leads to a colorimetric change of the PDA-DNA nanoparticles, which is affected by the concentration of MC-LR. To verify the analytical system, the pH-responsive performance of PDA-DNA was first investigated. We tested the PDA-DNA nanoparticles at various pH levels (ranging from 7.4 to 10) in the absence of MC-LR to simulate the final pH conditions that would result from urease-catalyzed urea hydrolysis. The UV–vis spectra of the nanoparticles were gradually changed as the pH increased, with significant shifts occurring between pH 8.5 and 10, corresponding to the expected pH range after urea hydrolysis (Fig. S7). The CR% of PDA-DNA caused by the pH stimulation was calculated using the equation:

$${CR\%}= \frac{PB_{b}-PB_a}{PB_b}{\%}$$

where *PB* = ${~}^{A_{blue}}\!\left/ \!{~}_{(A_{blue}+A_{red})}\right.$. $A_{blue}$ is the absorbance at 650 nm (for the blue phase), and $A_{red}$ is the absorbance at 540 nm (for the red phase). $PB_{a}$ and $PB_{b}$ are the *PB* values after and before the stimulation [31].

The CR% of PDA-DNA caused by pH 9 solution was found to be 45%, suggesting that the PDA-DNA system has a satisfactory response to pH variation. It also illustrates that the final pH of the urea hydrolysis is above 9, validating the use of this system for detecting MC-LR through pH-induced PDA transitions.

**Fig. 2:**
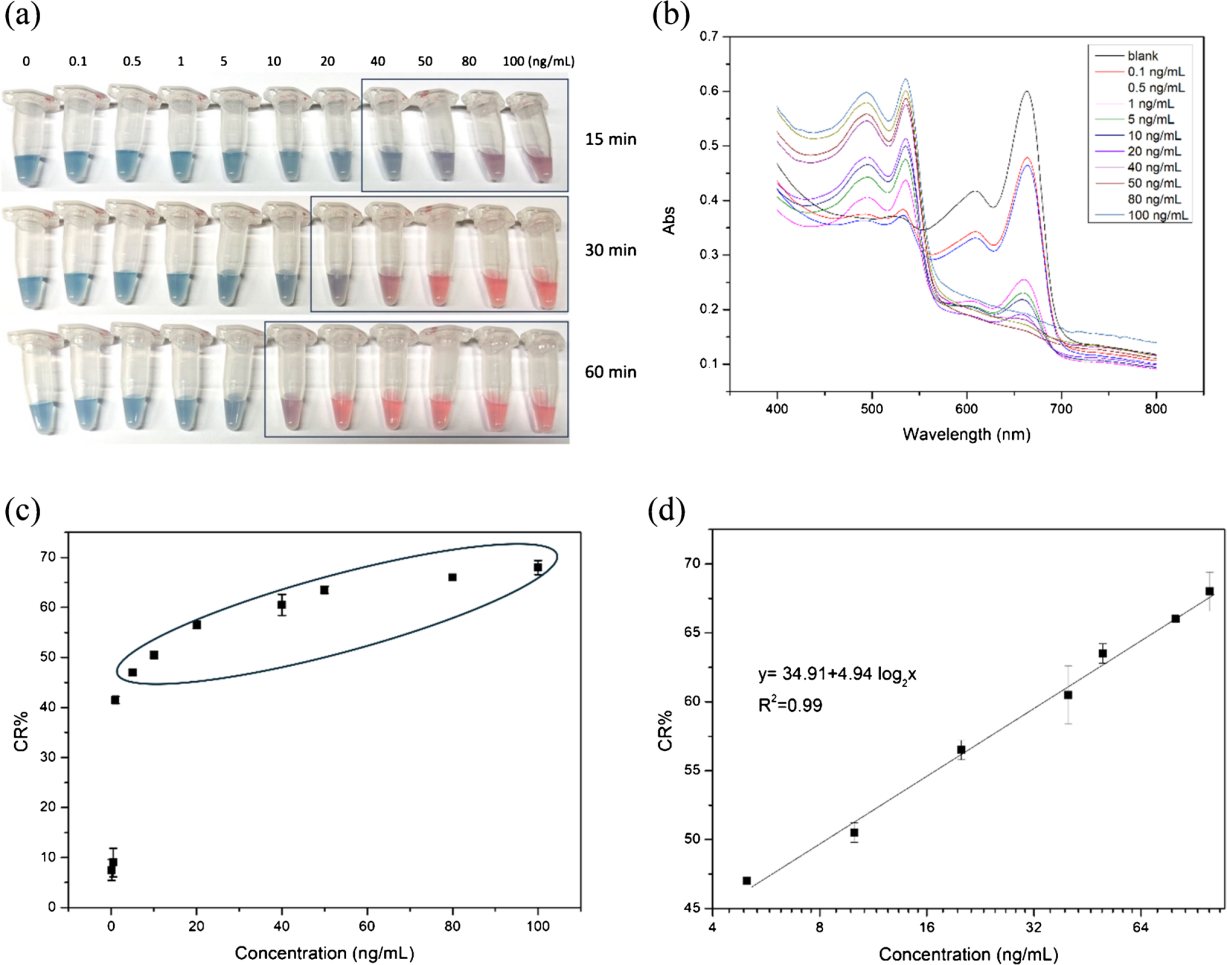
**a** The color of the PDA-DNA complex gradually changed from blue to red as the concentration of MC-LR increased with varying time (from 15 to 60 min). **b** Absorbance spectra of the colorimetric reaction product caused by different concentrations of MC-LR. **c** The response curve of the detection system from 0.1 to 100 ng/mL of MC-LR. **d** Relationship in a certain concentration range of MC-LR (5–100 ng/mL) corresponding to the black circle in **c**

For detection of MC-LR, key experimental conditions, including PDA-DNA polymerization time and urea concentration, were optimized to 20 min and 100 mM, respectively (Fig. S8). Using the optimized conditions, the entire analytical system for MC-LR detection was tested using standard samples. As the concentration of MC-LR increased, the final color of the PDA-DNA complex gradually changed from blue to red (Fig. 2a). Notably, the urease hydrolysis takes time, and the reaction mixture changed color from blue to purple within 15 min. The color continued to change into pink and red after 60 min, especially when tested against higher concentrations of MC-LR. Based on the change of UV–Vis spectrum over time (Fig. S9), we noticed that even at the highest concentration of MC-LR tested, the absorbance band at 650 nm ceased to change after 60 min. Consequently, the time for the urease reaction was set at 60 min in the final analytical method.

As shown in Fig. 2b, when MC-LR concentration increased in the range of 0.1–100 ng/mL, the absorbance at 650 nm continuously decreased and the absorbance at 540 nm increased. To quantify the degree of color change, we calculated the CR% caused by each MC-LR solution. With increasing MC-LR concentration, the CR% calculated from the absorbance spectra increased in proportion. A linear relation between CR% and MC-LR concentration was observed in the range of 5–100 ng/mL of MC-LR (y = 34.91 + 4.94log_₂_x, *R*^2^ = 0.99, *n* = 3). The limit of detection (LOD) was determined to be 1 ng/mL using the 3σ/slope definition (Fig. 2c). To achieve accurate quantification, a spectrophotometer can be used, but the color changes are also visible to the naked eye, as shown in Fig. 2a, with a LOD of 10 ng/mL. This LOD signifies the method’s potential for detecting MC-LR in environmental samples, emphasizing its sensitivity and practical utility.

### Selectivity and real sample analysis

**Fig. 3:**
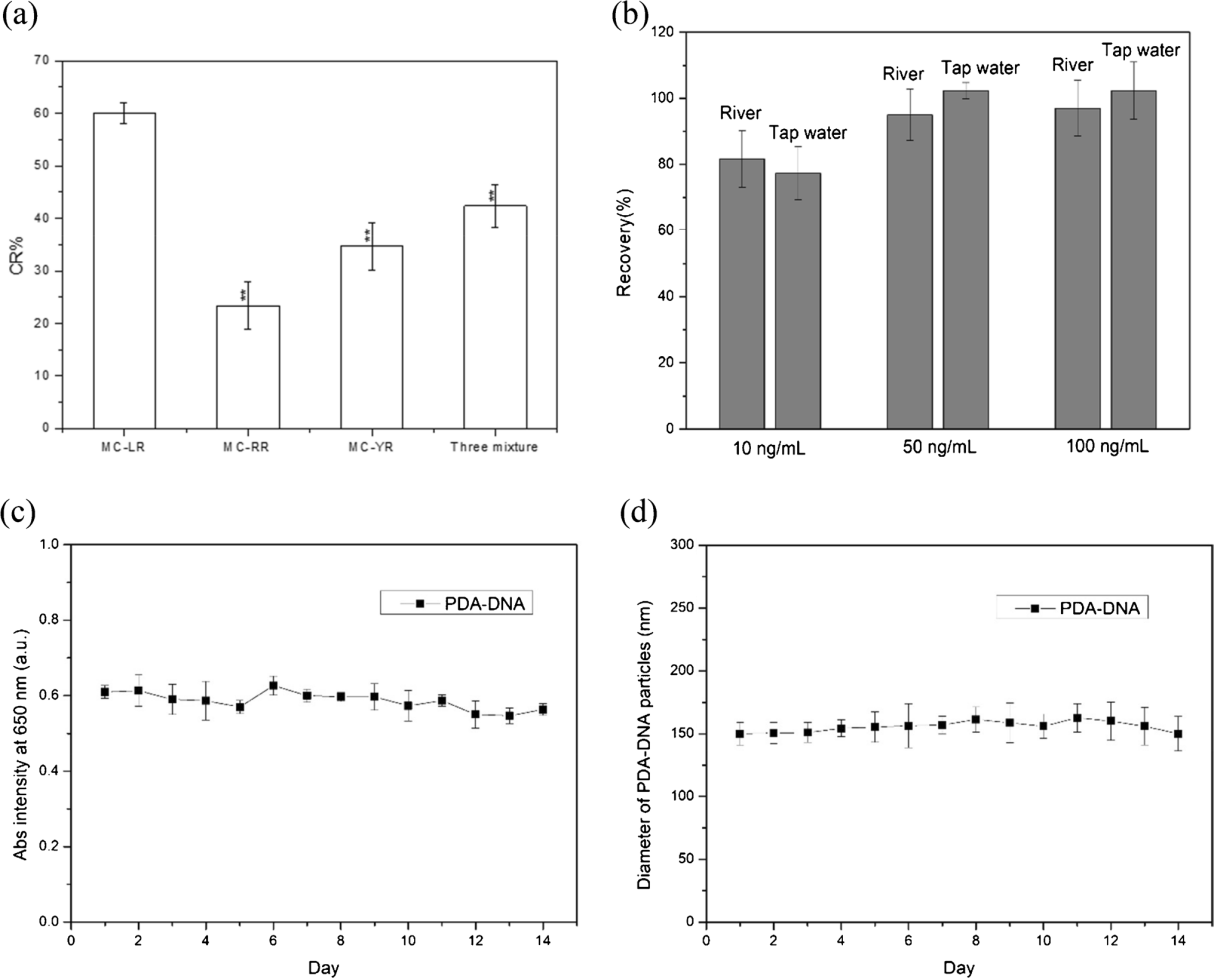
**a** Selectivity of PDA-DNA for the detection of MC-LR. The symbol ** means *p*-value < 0.001. **b** Real sample test at three different concentrations. **c** Absorbance intensity at 650 nm was measured every day for 2 weeks. **d** Diameter of PDA-DNA particles was measured every day for 2 weeks. Data represent mean ± SD (*n* = 3)

To assess the selectivity of our analytical system for MC-LR detection, we selected MC-RR and MC-YR as reference targets. As shown in Fig. 3a, the strongest detection signal was observed from MC-LR when it was tested at the same concentration (50 ng/mL), whereas the detection signals from MC-RR, MC-YR, and their mixture were weaker. This is because the aptamer exhibited high selectivity toward MC-LR over MC-RR and MC-YR. While all three microcystin variants are cyclic heptapeptides, they differ in their amino acid composition: MC-LR contains leucine (L) and arginine (R), MC-RR contains two arginine residues, and MC-YR contains tyrosine (Y) and arginine. The high affinity of the aptamer for MC-LR is primarily due to the hydrophobic interaction of leucine with the aptamer’s binding pocket. In contrast, the double arginine residues in MC-RR cause stronger electrostatic repulsion, reducing binding efficiency. Additionally, the bulkier tyrosine residue in MC-YR introduces steric hindrance, making it difficult for the aptamer to bind. As a result, the aptamer demonstrates significantly higher selectivity for MC-LR compared to MC-RR and MC-YR, ensuring accurate detection of MC-LR in environmental samples without interference from other variants. We also performed ANOVA and obtained an *F*-value of 46.66 and a *p*-value < 0.001, which provides strong evidence that there are significant differences between the CR% values of the different groups. The subsequent Tukey HSD test also clarified that each group pairs are statistically different from each other. Therefore, the analytical system exhibits good selectivity for MC-LR detection. To validate the reliability and applicability of the analytical system for detecting MC-LR in real samples, standard recovery experiments were conducted using river water and tap water spiked with varying concentrations of MC-LR (10 ng/mL, 50 ng/mL, 100 ng/mL). Figure 3b illustrates the effective detection of MC-LR in spiked water samples, achieving recovery rates between 81.6 and 97% for river water, and 77.3 to 102.3% for tap water. These results demonstrate the reliability and applicability of the method for real-world applications. In addition, we also studied the stability of the PDA-DNA nanoparticles. As shown in Fig. 3c, d, after 2 weeks of storage, the PDA-DNA nanoparticles did not exhibit noticeable changes in particle size, and the absorbance intensity at 650 nm remained unchanged. These results confirm that the PDA-DNA nanoparticles are stale without agglomeration, and their colorimetric properties are maintained. Finally, compared to the analytical performance of previously reported colorimetric assays, the present method for MC-LR detection demonstrates good sensitivity and a more straightforward colorimetric readout (Table S2). Our colorimetric approach not only achieves a low-cost method but also allows for visual detection, making it more accessible for field applications.

## Conclusion

In this study, we developed a novel nucleic acid biosensor system using polydiacetylene for the sensitive detection of MC-LR. The system utilizes magnetic beads conjugated with an MC-LR aptamer and cDNA-urease to respond to MC-LR, where the target binding triggers a conformational change of the aptamer to release the cDNA-urease from the magnetic beads. The released cDNA-urease is then captured by a DNA-functionalized PDA to catalyze hydrolysis of urea to change the color of the PDA-DNA nanoparticles. Our detection system demonstrated good sensitivity, with a linear response range between 5 and 100 ng/mL and a limit of detection as 1 ng/mL. The ability to visually observe the color change underscores the practical applicability of this biosensor for field detection. Selectivity tests against other microcystin variants (MC-RR and MC-YR) and mixtures confirmed the specificity of our system for MC-LR detection. The combination of PDA and nucleic acid in our biosensor presents a powerful approach for detecting low molecular weight analytes, overcoming the limitations of existing PDA-based nucleic acid sensing methods. Also, this work addresses the limitations of existing techniques by providing a low-cost, user-friendly alternative that can effectively discriminate MC-LR in the presence of structurally similar microcystins, thereby advancing the field of environmental monitoring. Future research will aim to enhance the biosensing platform by enabling simultaneous detection of multiple targets within a single system, and further development of assay kits. The results of this study pave the way for advanced biosensing technologies with potential applications in environmental monitoring and public health.

## Supplementary Information

Below is the link to the electronic supplementary material.

Supplementary file1 (PDF 1122 KB)
