# Supplementary material for: Colorimetric aptasensing of microcystin-LR using DNA-conjugated polydiacetylene
Source: Anal Bioanal Chem. 2024 Oct 28;416(29):7131–40. doi: 10.1007/s00216-024-05617-x (PMC11579182; doi:10.1007/s00216-024-05617-x)
Supplement: Supplementary file 1 — Supplementary file1 (PDF 1122 KB) [file 216_2024_5617_MOESM1_ESM.pdf]

Supporting Information

Colorimetric aptasensing of microcystin-LR using DNA-conjugated polydiacetylene

Man Zhang, Qicheng Zhang, Lei Ye*

Division of Pure and Applied Biochemistry, Department of Chemistry, Lund University, 22100 Lund, Sweden

*Corresponding author email: lei.ye@tbiokem.lth.se

(a)

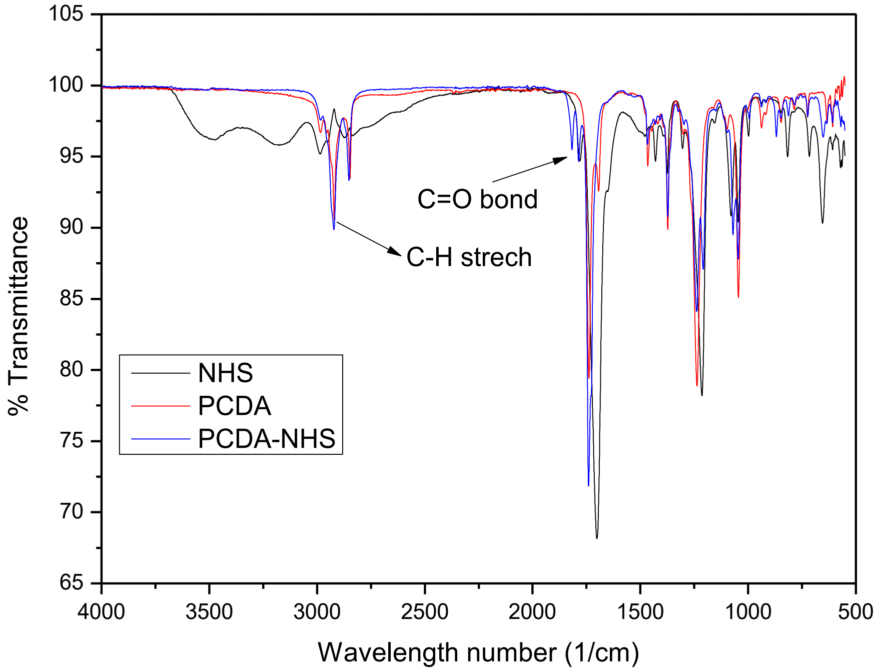

(b)

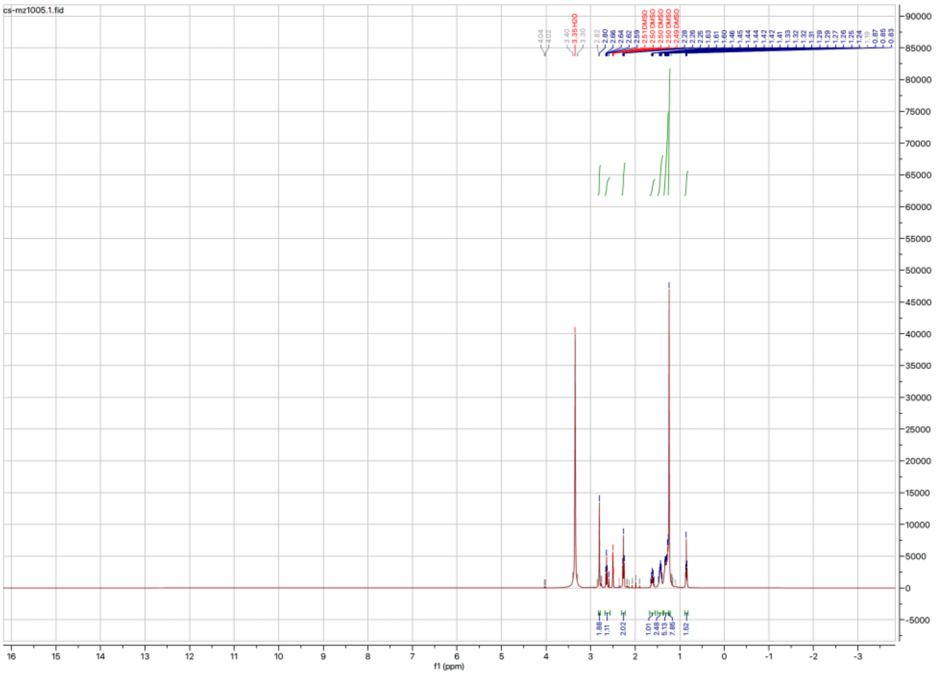

Fig. S1 (a) FT-IR spectra of PCDA (green) and PCDA-NHS (red). The FT-IR spectrum of PCDA shows unique carboxylic acid (-COOH) stretching signal at 1690 cm^{-1}. This carboxylic acid signal is absent in the PCDA-NHS spectrum. (b) ^{1}H-NMR spectrum of PCDA-NHS. The NMR spectrum (500 MHz) was obtained from 20 mg of PCDA-NHS dissolved in 1 mL DMSO-*d6*.

(a)

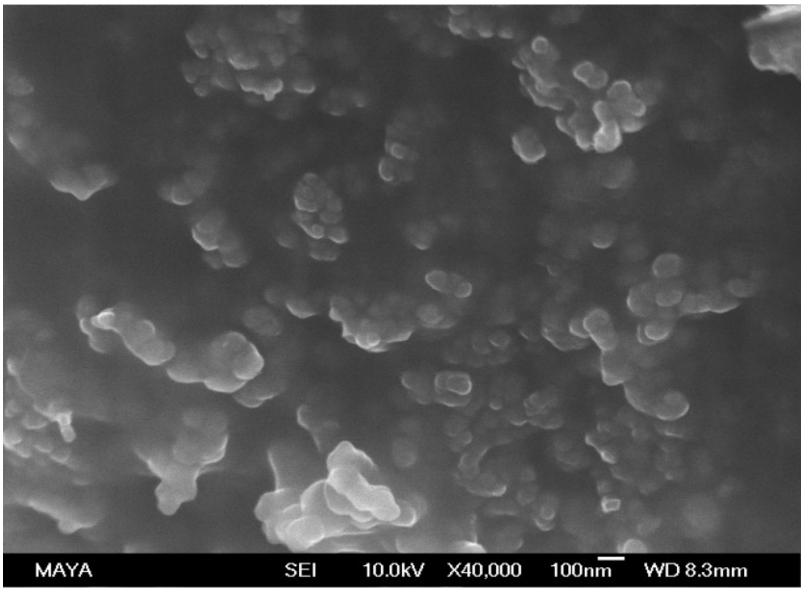

(b)

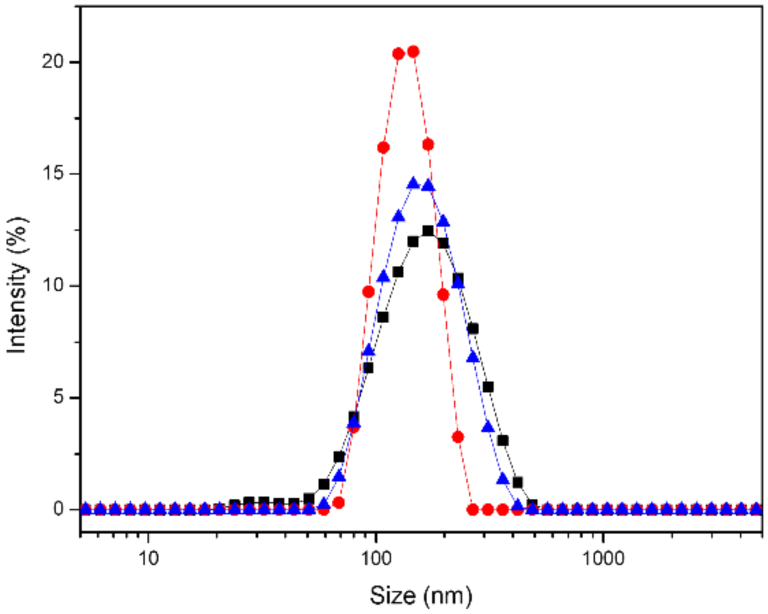

Fig. S2 (a) SEM image and (b) hydrodynamic size for PDA particles prepared without DNA modification.

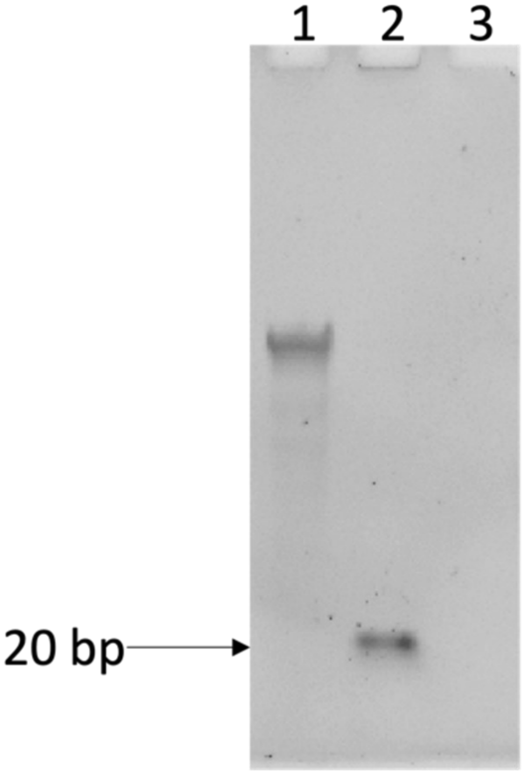

Fig. S3 12% PAGE gel image. Lane 1: PDA-DNA (approx.1 μM). Lane 2: Free DNA (1 μM). Lane 3: PDA particles (1 mM).

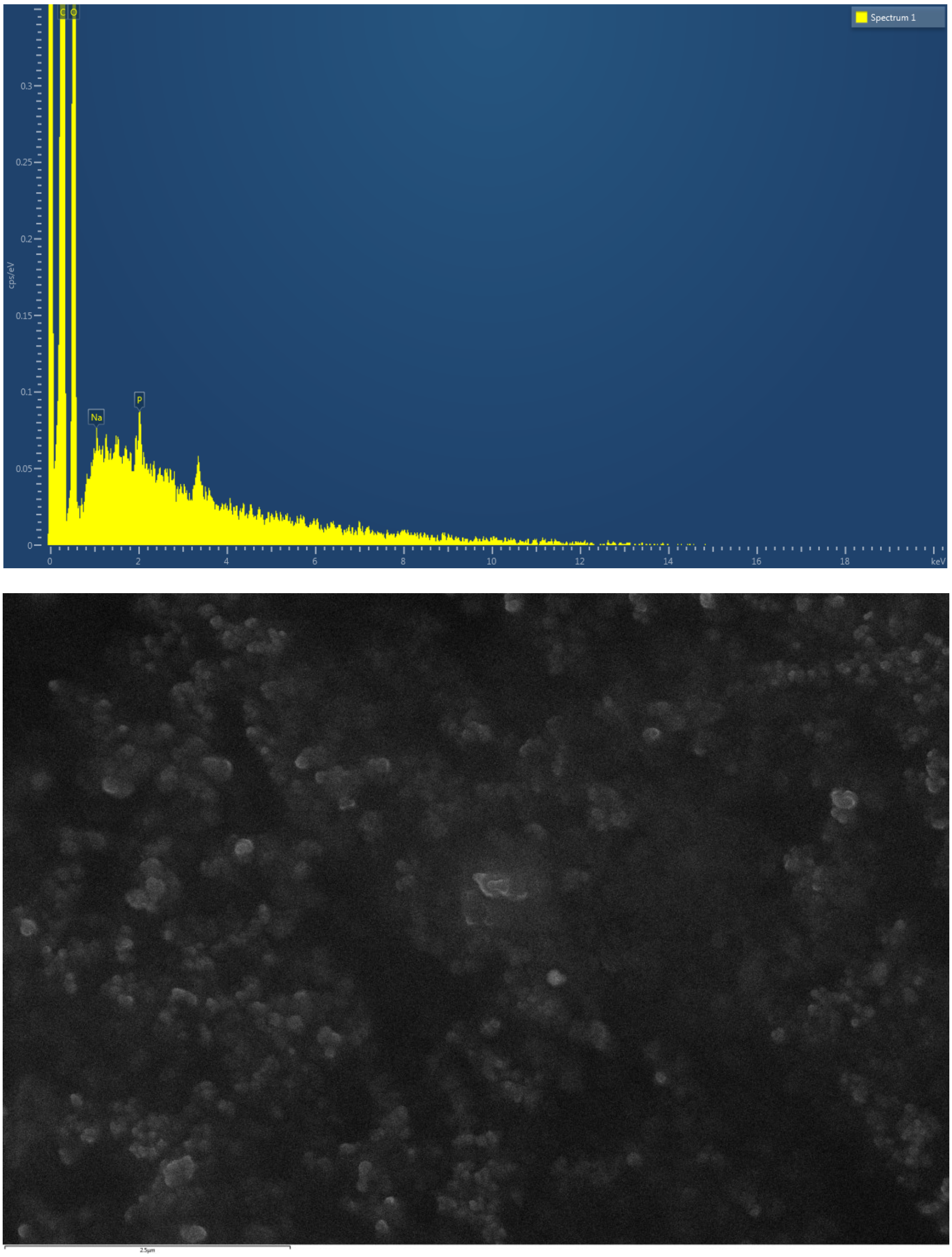

Fig. S4 Energy-dispersive X-ray spectroscopy (EDS) image of PDA-DNA. The presence of the phosphorus element in the PDA-DNA sample confirmed the successful conjugation.

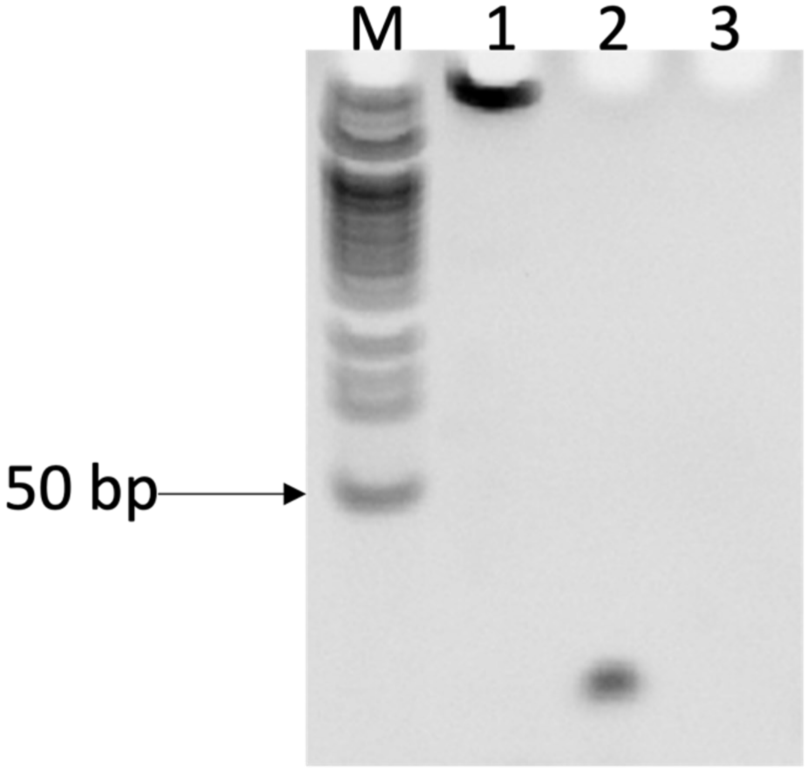

Fig. S5 12% PAGE gel image. M: Marker. Lane 1: Purified urease-DNA (approx.10 μM). Lane 2: Mixture of urease and NH_2-DNA (10 μM). Lane 3: Urease (1 mg/mL).

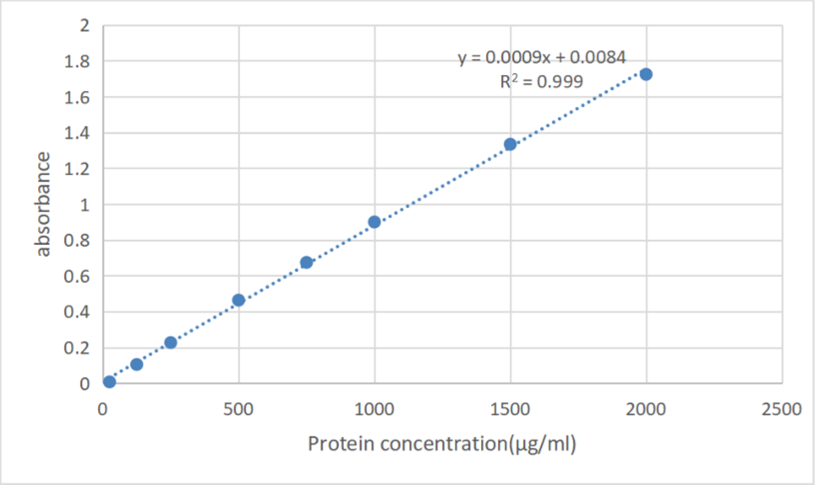

Fig. S6 Standard curve for determination of protein concentration. After the conjugation reaction, the absorbance of cDNA-urease at 562 nm was found to be 0.516. Using the linear regression equation, the protein concentration was calculated as 0.554 mg/mL. Based on the initial concentration of urease used (1.5 mg/mL), the conjugation efficiency was 37%.

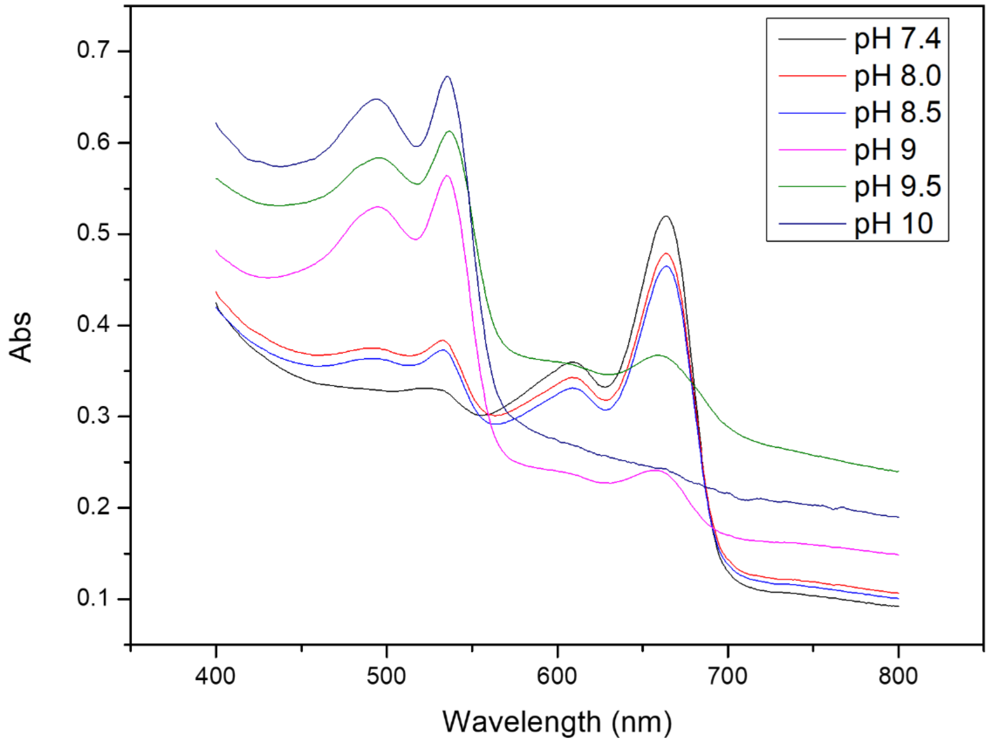

Fig. S7 UV-vis spectra of PDA-DNA nanoparticles after stimulation with solutions of different pH values. PDA-DNA (100 μL, 10 mM) was mixed with 100 μL solution of different pH at room temperature for 30 min before the visible absorbance spectrum was measured.

(a)

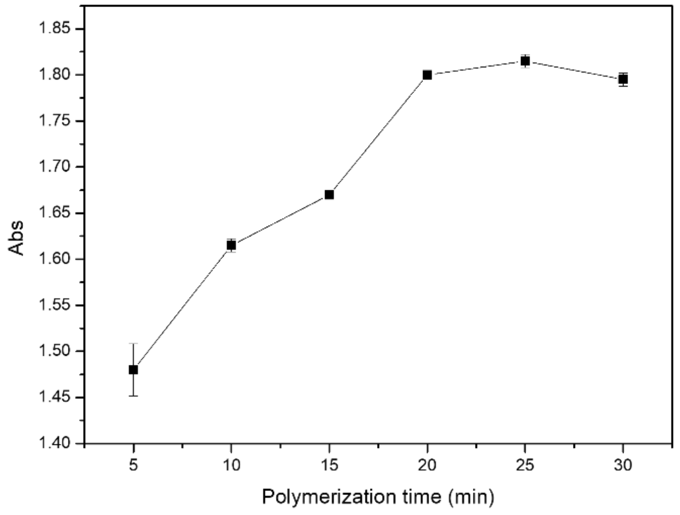

(b)

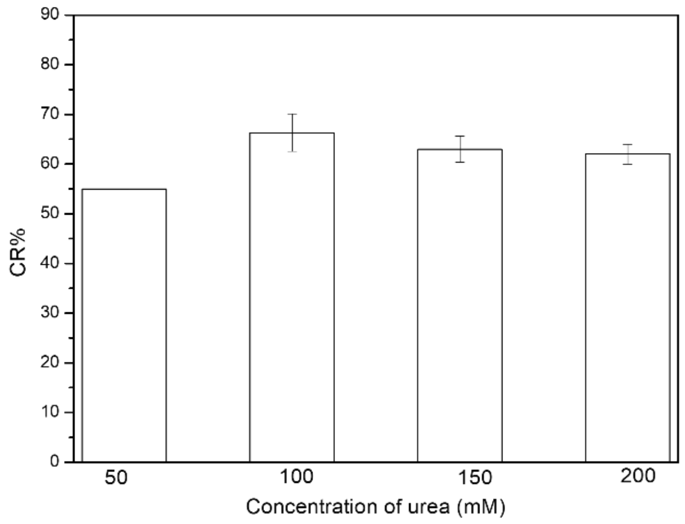

Fig. S8 Optimization of (a) polymerization time and (b) the concentration of urea. The optimization experiments were under the conditions as: (a) The stock solution (10 mM) was polymerized under UV (254 nm) for different before absorbance intensity of the sample was measured at 650 nm. (b) Urea solution (100 μL) at different concentrations was added to a mixture composed of 100 μL PDA-DNA (10 mM) and 50 μL cDNA-urease containing 50 ng/mL MC-LR at room temperature and reacted for 60 min prior to measuring the CR%.

(a)

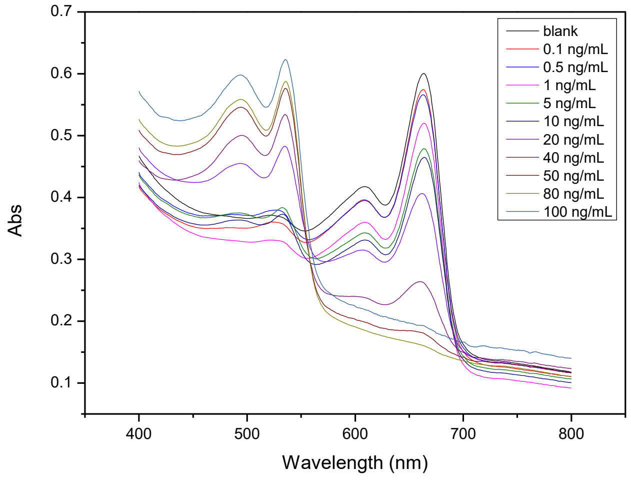

(b)

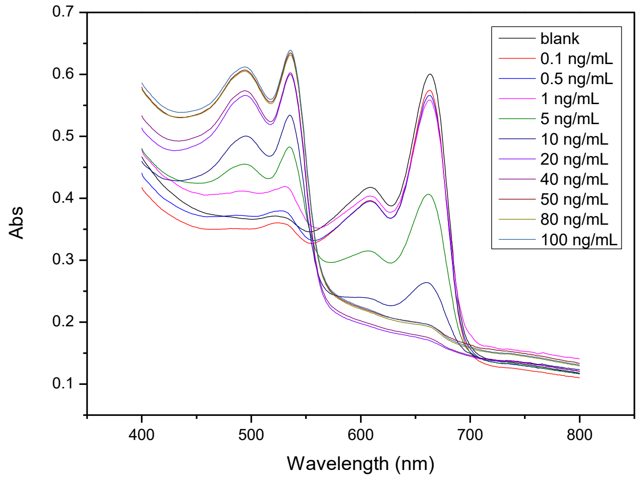

(c)

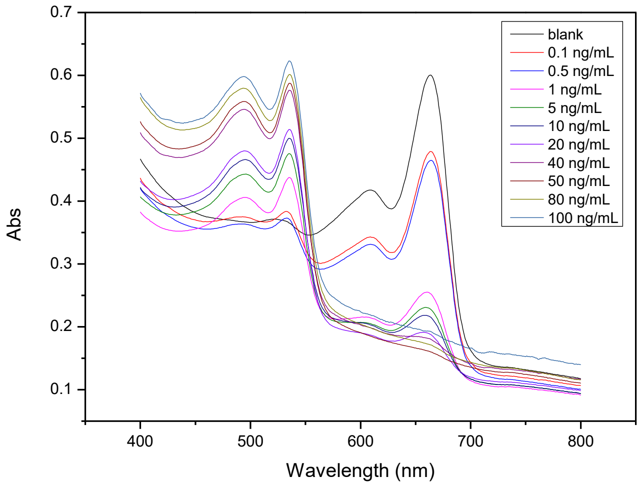

Fig. S9 UV-vis spectra for the detection of MC-LR after 15 min (a), 30 min (b) and 60 min (c).

Table S1 Nucleic acid sequences used in this work.

Name	Sequence (5'-3')
MC-LR aptamer	Biotin-GGCGCCAAACAGGACCACCATGACAATTACCCATACCACCTC ATTATGCCCCATCTCCGC
cDNA	NH_2-GAT GGG GCA TAA TGA GGT GG
NH_2-DNA	NH_2-CCA CCT CAT TAT GCC CCA TC

Table S2 Comparison of different systems for MC-LR colorimetric detection.

Detection system	Method	Limitation of detection	References
Magnetic particle based enzyme assays	Colorimetric	7.4 ng/mL	[1]
Antibody immunoassay	Colorimetric	1.8 ng/mL	[2]
Aptamer-based microcantilever array	Colorimetric	1.1 ng/mL	[3]
Polydiacetylene and antibody	Colorimetric	1.0 ng/mL	[4]
Polydiacetylene-DNA and magnetic-aptamer	Colorimetric	1.0 ng/mL	This work

References

1. Reverte, Laia, et al. Magnetic particle-based enzyme assays and immunoassays for microcystins: from colorimetric to electrochemical detection. Environ. Sci. Technol. 2013;47(1):471-478.
2. Sheng, Jian-Wu, Miao He, and Han-Chang Shi. A highly specific immunoassay for microcystin-LR detection based on a monoclonal antibody. Anal. Chim. Acta 2007; 603(1):111-118.
3. Zhang, Guangping, et al. Label-free aptamer-based detection of microcystin-LR using a microcantilever array biosensor. Sens. Actuators, B 2018;260:42-47.
4. Xia, Yuetong, Jieli Deng, and Long Jiang. Simple and highly sensitive detection of hepatotoxin microcystin-LR via colorimetric variation based on polydiacetylene vesicles. Sens. Actuators, B 2010;145(2):713-719.
